# Identification of a Potential Third Component of the Male-Produced Pheromone of *Anoplophora glabripennis* and its Effect on Behavior

**DOI:** 10.1007/s10886-014-0520-3

**Published:** 2014-11-29

**Authors:** Damon J. Crook, David R. Lance, Victor C. Mastro

**Affiliations:** Otis Laboratory, USDA APHIS PPQ CPHST, 1398 West Truck Road, Buzzards Bay, MA 02542-1329 USA

**Keywords:** Pheromone, Invasive species, Cerambycidae, (3*E*,6*E*)-α-farnesene

## Abstract

The Asian longhorned beetle, *Anoplophora glabripennis*, is considered to be one of the most serious invasive pests of deciduous trees in North America. An efficient monitoring trap is needed to detect and delimit new introductions and assess population densities of established infestations. Previous studies on *A. glabripennis* have shown that males produce a two-component aggregation pheromone that consists of a 1:1 blend of 4-(*n*-heptyloxy)butan-1-ol and 4-(*n*-heptyloxy)butanal. Moderate attraction in field trapping studies suggested that there may be additional chemical cues missing. Volatiles from male *A. glabripennis* were examined to identify other potential pheromone components. Gas chromatographic / electroantennographic (GC/EAD) analyses of male aerations detected a consistent EAD-active response to a previously unidentified compound. This compound was identified as (3*E*,6*E*)-α-farnesene. Both male and female beetles were antennally responsive to this sesquiterpene, and both sexes were attracted to it in olfactometer bioassays at different doses. When (3*E*,6*E*)-α-farnesene was combined with 4-(*n*-heptyloxy)butan-1-ol and 4-(*n*-heptyloxy)butanal, attraction of both sexes increased compared to assays using 4-(*n*-heptyloxy)butan-1-ol and 4-(*n*-heptyloxy)butanal alone.

## Introduction

The Asian longhorned beetle, *Anoplophora glabripennis* (Motschulsky) (Coleoptera: Cerambycidae: Lamiinae) is a serious wood-boring pest of hardwood trees that has been introduced from China to North America and Europe in wood packaging materials (Nehme et al. 2010). In North America, it was first detected in 1996 in Brooklyn, New York. Since then infestations from separate introductions have been found in Illinois (1998), New Jersey (2003), Toronto (2003), Massachusetts (2008), and Ohio (2011) (Wickham et al. 2012). Despite *A. glabripennis* having a host range of over 43 hardwood trees, North American populations favor six species of maple, *Acer* spp. (Haack et al. 1996, 2010; Hoover et al. 2014; Hu et al. 2009; Morewood et al. 2004). This preference for maple could have a drastic impact on lumber and maple syrup industries, as well as the urban forests of North America (Nehme et al. 2010). Up to 1.2 billion urban shade trees (worth up to $669 billion) are at risk if *A. glabripennis* becomes well established in the U.S. (Nowak et al. 2001).

For current U.S. infestations, management is based on detection, tree removal, and insecticidal control (Wickham et al. 2012). Monitoring for *A. glabripennis* is both labor intensive and expensive, involving climbing trees and searching for oviposition scars, larval frass, sap flow, and emergence holes (Nehme et al. 2010). An attractant-baited trap that is capable of detecting low level populations could greatly improve the efficiency and cost-effectiveness of *A. glabripennis* surveys. Chemical attractants also could potentially be used in combination with biological control agents such as fungal pathogens specific to *A. glabripenni*s (Dubois et al. 2004a, b; Hajek et al. 2006).

Several studies have shown that mate-finding in *Anoplophora glabripennis* involves numerous chemical cues and behaviors, although the exact sequence of events remains unclear (Hoover et al. 2014; Wickham, et al. 2012). Male *A. glabripennis* produce a blend of two dialkylethers, 4-(*n*-heptyloxy)butan-1-ol and 4-(*n*-heptyloxy)butanal (Zhang et al. 2002). These compounds elicit GC-EAD responses in females, and are moderately attractive in laboratory assays, particularly when used with host odors such as (−)–linalool, *trans*-caryophyllene, and (*Z*)-3-hexen-1-ol (Meng et al. 2014; Nehme et al. 2009; Zhang et al. 2002). When these two male-produced compounds were used in field bioassays in China (Ningxia Province), captures of *A. glabripennis* were significantly greater on baited traps than unbaited controls (Meng et al. 2014; Nehme et al. 2010). Wickham et al. (2012) stressed that current pheromone blends appear to be incomplete, and that identification of further pheromone components may lead to an ‘operational-ready’ lure for monitoring *A. glabripennis* effectively at low population densities. Here, we report the identification of (3*E*,6*E*)-α-farnesene, as a potential third pheromone component produced by males of *A. glabripennis*. We also demonstrate attraction of adult male and female beetles to (3*E*,6*E*)-α-farnesene in laboratory assays.

## Methods and Materials

### Insects

All male adults used for aerations in 2012 and 2013 emerged from infested wood of red maple, *Acer rubrum* L., that were collected in and around Bethel, Ohio, and transported under permit to the USDA APHIS PPQ CPHST insect containment facility in Buzzards Bay, MA, USA.

### 2012 Aerations

Insects were reared using similar protocols to those of Zhang et al. (2002). Adult virgin beetles were collected daily from infested logs before being kept individually in 2.1 L plastic jars (Rez-Tech Corporation, OH, USA). Three small holes in the lid of each jar provided ventilation. Insects were fed several approx 16 cm twigs of striped maple *Acer pensylvanicum L.* The twigs and a 4 × 1 cm cotton dental wick extended through holes in the lid of a water-filled 120 ml plastic cup (Dart Corporation, MI, USA) in the bottom of each jar. A coffee filter (8–12 cup size) was placed in the bottom of each jar to collect frass and absorb spilled water. Jars holding males and females were held in the same environmental chamber at 25 °C, approx. 60 % relative humidity (RH) and 16:8 h L:D.

Lighting was provided by three standard fluorescent bulbs (4100 k 32 watt, 300 lx) in clear plastic light box fittings. Insects were fed for 10 days before being used in aerations. Male beetles were aerated in an environmental chamber that contained no females or plant material. Individual 10-day-old virgin adults were placed in 120 ml glass canning jars (Bell/Uline, WI, USA) with Teflon screw-on lids that had two openings for tubing.

Battery operated pumps (Sensidyne, Clearwater, FL, USA) pulled air through each jar at a rate of 300 ml/min. Ambient air was filtered through a 6–14 mesh activated charcoal (Fisher Scientific, Pittsburgh, PA, USA) inlet before entering the jar. After leaving the jar, the air passed through two traps connected by a short section of Teflon tubing. Each trap was a 3 mm ID × 110 mm glass tube containing 200 mg of 50/80 mesh Super Q (Alltech, USA). All connections were sealed with Teflon® tape. Sampling was conducted at approximately 25 °C, approx 60 % RH and 16:8 h L:D using three standard fluorescent bulbs in clear plastic boxes (670 lx output). Three beetles, in separate jars, were aerated simultaneously. Once collected, aeration samples were eluted with 2 ml of CH_2_Cl_2_ (J. T. Baker, NJ, USA). The three male extracts then were pooled before being concentrated to 100 μl under a gentle stream of N_2_. All extracts were kept at −20 °C before GC/MS or GC/EAD analysis. Aerations lasted from 0900 h until 0900 h the next day (24 h). During 2012, 13 groups of 10-d-old virgin males (3 per group) were aerated. Nine virgin males between 15 and 23 days of age were also aerated and pooled into three samples (3 males each). Samples of headspace volatiles that contained the largest number of compounds (after examination by GC/MS) were used for GC/EAD recordings.

### 2013 Aerations

Based on the results from 2012, we decided to change several aspects of the adult holding protocols the following year. The plastic jars were replaced by 1.9 L glass canning jars (Bell/Uline, WI,) with 7.5 cm diam wire mesh lids. Insects were fed for 10 days before being used in aerations. Males and females were kept in separate rooms within the containment facility on shelving units that were screened from ambient lab lighting. ‘Laboratory’ lighting in 2013 was changed to approximate more closely the wavelengths and intensity of ‘natural’ sunlight, as cerambycid beetles have been reported to behave sedentary or agitated under laboratory light conditions (Lacey et al. 2004). Specifically, the light systems each consisted of four T5 fluorescent lamps (Deep Blue Professional, City of Industry, CA, USA). Two were 39-W Solarmax T5 10,000 K daylight lamps, and two were 39-W Solarmax T5 actinic 03 lamps that emitted a max blue phosphors peak at 420 nm. The regimes for these two lamp types were controlled by automatic timers and set as follows: the blue actinic lamps turned on at 0630 h and shut off at 2100 h; the two daylight bulbs turned on at 1030 h and switched off at 1530 h. This lighting setup allowed for a morning and late evening actinic lamp period with a bright daylight period through midday. Light output from the actinic bulbs alone was measured at 80 lx. Light output for the daylight and actinic lamps together was 450 lx. The light system was suspended 15–20 cm above the rearing/feeding jars for both male and female colonies. In 2013, male 10-day-old virgin adults were aerated using the same jars and pump system as in 2012, but collections were done under the new lighting system described above at 25 °C and approx 55 % RH. Adults were aerated without plant material being present. Collections were made between 1000 and 1500 h using a single Super Q cartridge of 200 mg and eluted with 1.5 ml of hexane (HPLC grade, OMNISOLV). Individual male collections were concentrated to 100 μl under a gentle stream of N_2_, and stored at −20 °C before GC/MS or GC/EAD analysis.

### GC/MS Analyses

Initial chemical analyses were conducted using a combined Agilent Technologies 6890 network gas chromatograph and 5973 mass-selective detector. The GC was equipped with a DB-5 column (30 m × 0.25 mm I.D.; film thickness, 0.25 μm; J & W Scientific Inc., Folsom, CA, USA). Helium was the carrier gas at a constant flow rate of 0.7 ml/min. Injection was splitless at 275 °C. Oven temperature was held at 50 °C for 2 min, programmed to 280 °C at 10 °C/min and held for 15 min. Volatiles were identified based on their mass spectra (NIST version 2.0, 2002), Kovats indices (Kovats 1965; Van Den Dool and Kratz 1963) and comparison of the retention indices and mass spectra with those of available authentic synthetic compounds. Two separate authentic standards of (3*E*,6*E*)-α-farnesene were obtained from the University of California, Riverside, CA, USA, and USDA-ARS, Beltsville, MD, USA. The (3*E*,6*E*)-α-farnesene from Riverside, CA, was used for GC/EAD analysis and was at least 86 % pure based on peak area values by GC. A sample of (3*Z*,6*E*)-α-farnesene (containing 0.1 % BHT) was obtained from the Natural Resources Canadian Forest Service laboratory (Fredericton, NB, Canada) and was at least 90 % pure based on the peak area values by GC. A mixture of farnesene isomers was obtained from Sigma Aldrich Co. (St Louis, MO, USA). Synthesis methods for (3*E*,6*E*)-α-farnesene and (3*Z*,6*E*)-α-farnesene are described by Khrimian et al. (2012) and Silk et al. (2010). The alcohol 4-(*n*-heptyloxy) butan-1-ol and aldehyde 4-(*n*-heptyloxy) butanal were obtained from Bedoukian Research Inc. (Danbury, CT, USA).

### Electrophysiological Analysis (GC/EAD)

The coupled GC/EAD system used was as previously described by Crook et al. (2008) with a few modifications. Samples of aerations or standards (2 μl) were injected in splitless mode onto a Hewlett Packard (Agilent) 6890 gas chromatograph with a DB-5MS-DG column (30 m × 0.25 mm ID, 0.25 μm film thickness; J & W Scientific Inc.) and a 1:1 effluent splitter that allowed simultaneous FID and EAD detection of the separated volatile compounds. Helium was the carrier gas (2.5 ml/min). Oven temperature was held at 50 °C for 2 min, programmed to 280 °C at 10 °C/min and held for 15 min. Injector temperature was 275 °C. The GC outlets for the EAD and FID were 280 °C.

The column outlet for the EAD was held in a water-cooled humidified air stream (20 °C) flowing at 2 ml/min over the prepared antennae of adult *A. glabripennis* attached to an EAG probe (Syntech, Hilversum, the Netherlands). Antennae were prepared by cutting a single antenna at the base of the head of an adult beetle, and removing the lower pedicel and scape. A size 1 insect pin (BIOQUIP®) was used to make three holes on the first flagellomere as well as the flagellomere third from the tip. Holes were made deep enough to make a clean opening in the cuticular surface to allow conducting gel (Spectra 360, Parker Laboratories, Fairfield, NJ, USA) to form an uninterrupted connection to the EAG probe. One of the electrodes on the probe was extended with gold wire (20 mm long) to accommodate the long length of the antennal preparation. This method preserved the tip of the antennae, eliminating the risk of removing vital sensillae specifically located there (Crook et al. 2003). The EAG probe was connected to an IDAC-232 serial data acquisition controller (Syntech). Signals were stored and analyzed on a PC equipped with the program EAD (version 2.6, Syntech).

### Olfactometer Assays

A Y-tube olfactometer (Analytical Research Systems Inc., Gainsville, FL, USA) was used to test biological activity of synthetic samples. All behavioral assays were done in a walk-in environmental chamber (25 °C, approx 60 % RH) under a lighting system (4 × T5 Solarmax fluorescent lamps) described earlier. The Y-tube was held at a 15° angle upward from horizontal on a custom built holder placed 0.5 m below the lighting (measured at 300 lx). The glass tube (3.5 cm internal diam) had a 15 cm main stem that branched into two 13 cm arms angled at 90 °. Each arm then was connected to a separate glass tube that contained the stimulus or a solvent/blank control. Charcoal filtered air was bubbled through distilled water and then into each of the two arms at 1.0 l/min using a 2-channel air delivery system (Analytical Research Systems Inc., Gainesville, FL, USA).

Male and female adults between 15 and 40-day-old were used for all olfactometer bioassays. Individual beetles used in these bioassays were from 2013 colonies (see above). Insects were feeding on twigs until used in tests, *i.e.*, with no starvation period. A total of 25 replicates was completed for each treatment, using one beetle per replicate.

Stock solutions of (3*E*,6*E*)-α-farnesene (CA sample), 4-(*n*-heptyloxy)butan-1-ol and 4-(*n*-heptyloxy)butanal (10 μg per 10 μl hexane) were used for all tests.

Bioassays were conducted to test attraction to (3*E*,6*E*)-α-farnesene either alone (1 μg, 10 μg, and 20 μg levels) or in combination (1:1:1) with 4-(*n*-heptyloxy)butan-1-ol and 4-(*n*-heptyloxy)butanal (at the 1 μg and 10 μg concentrations). These doses were selected based on previous olfactometer bioassays involving *A. glabripennis* (Nehme et al. 2009; Zhang et al. 2002). Treatments were offered against a hexane control. A 1:1 component mix of 4-(*n*-heptyloxy)butan-1-ol and 4-(*n*-heptyloxy)butanal also was tested at the 10 μg concentration against a hexane control. The test stimulus was dispensed onto a strip of filter paper (10 × 40 mm), and placed in the tube connected to one arm of the olfactometer. An identical filter paper strip with the same amount of hexane was placed in the other arm of the olfactometer. The Y-tube was rinsed with acetone between each individual test. Treatment and control arms were alternated every other replicate to control for possible positional effects. For each test, a single male or female beetle was placed at the end of the main stem and given 5 min to choose between the two stimuli. A choice was recorded when the beetle passed a line, 8 cm beyond the branch point of each arm. No choice was recorded if the beetle failed to pass either line after the 5 min period. Insects that did not make a choice were excluded from statistical analysis. Out of 388 olfactometer tests only 13 beetles failed to make a choice (3.35 %). All experiments were conducted between 1100 and 1500 h, when beetles appeared to be most active.

### Statistical Analyses

To test whether the test stimulus attracted more beetles than solvent control in Y-tube olfactometer bioassays, a *X* ^2^ analysis goodness-of-fit test was used. Values of *X* ^2^ > 3.84 with 1 *d.f*. were significant at α = 0.05 (Epistat 2.1, TX, 1983).

## Results

### Identification of Male-Specific Chemicals

**Fig. 1:**
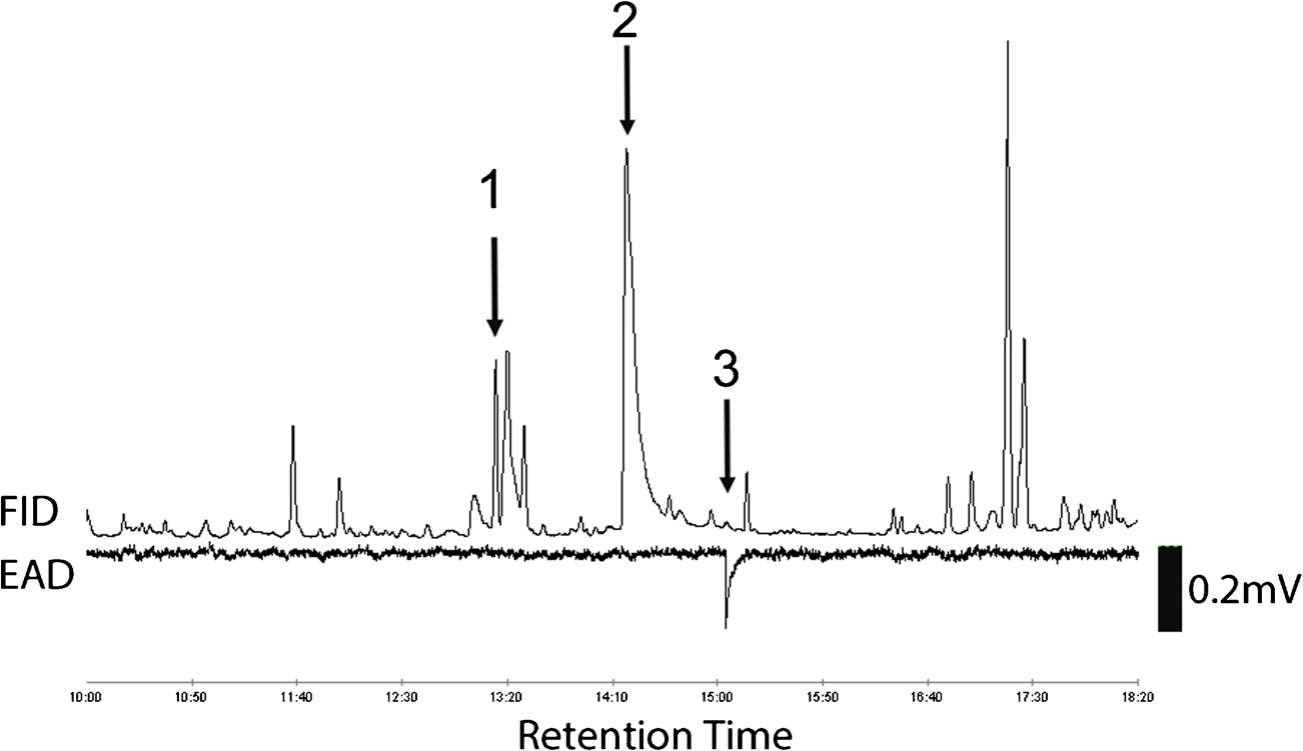
GC/EAD response of 2-day-old female *Anoplophora glabripennis* to a male aeration sampled in 2012 ((*1*) 4-(*n*-heptyloxy)butanal; (*2*) 4-(*n*-heptyloxy)butan-1-ol; (*3*) unknown)

**Fig. 2:**
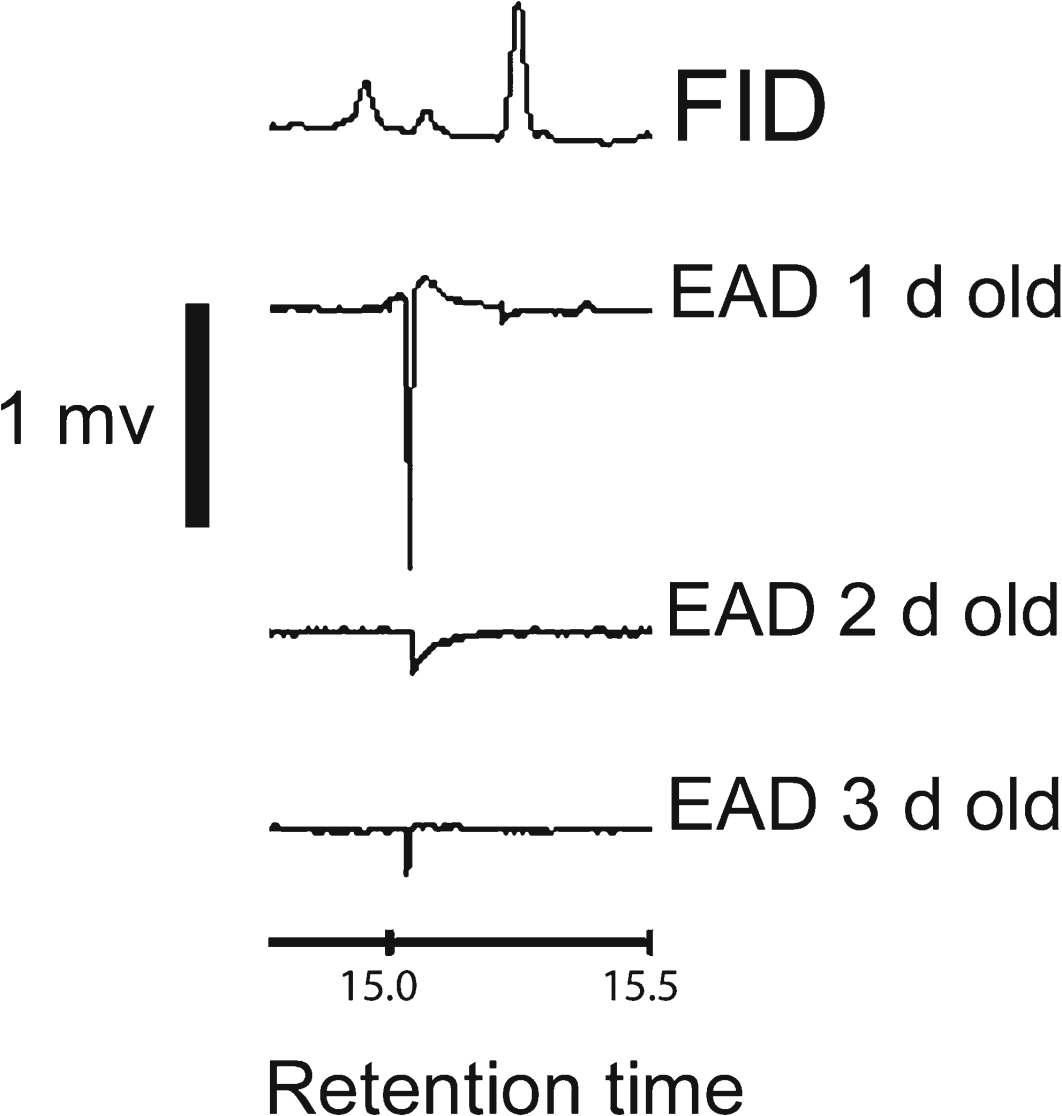
EAD responses of 1, 2, and 3-day-old fed virgin female *Anoplophora glabripennis* to unknown peak in male aeration sample from 2012

Aerations completed during 2012 contained the two male-produced compounds, 4-(*n*-heptyloxy)butanal and 4-(*n*-heptyloxy)butan-1-ol, previously reported by Zhang et al. (2002). Of these two compounds, 4-(*n*-heptyloxy)butanal did not elicit a consistent antennal response during GC/EAD tests. Occasional antennal responses were observed to the 4-(*n*-heptyloxy)butan-1-ol from male aeration samples passed over female antennae . A consistent GC/EAD response from females was found for a trace peak (retention time 15.1 min), from several pooled samples of aerations collected from males during 2012 (Fig. 1). The peak at 15.1 min was absent from aeration samples of females (fed and unfed) or striped maple twigs used in rearing. This compound elicited responses from the antennae of virgin female *A. glabripennis* that had been fed twigs for 1, 2, and 3 days (Fig. 2), and from the antennae of 14-day-old fed virgin females (data not shown).

**Fig. 3:**
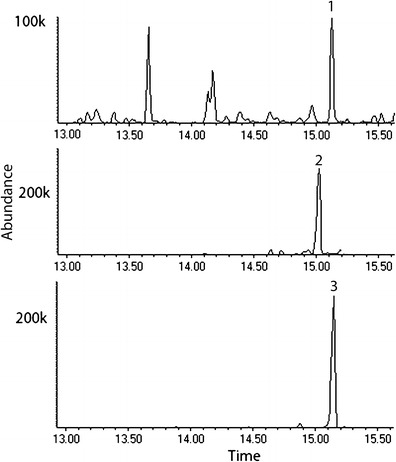
Total ion chromatogram of (*1*) unidentified peak in GC/MS analysis of aeration sample from male *Anoplophora glabripennis* collected under Solarmax T5 lighting system in 2013; (*2*) synthetic standard of (3*Z*,6*E*)-α-farnesene; (*3*) synthetic standard of (3*E*,6*E*)- α-farnesene

**Fig. 4:**
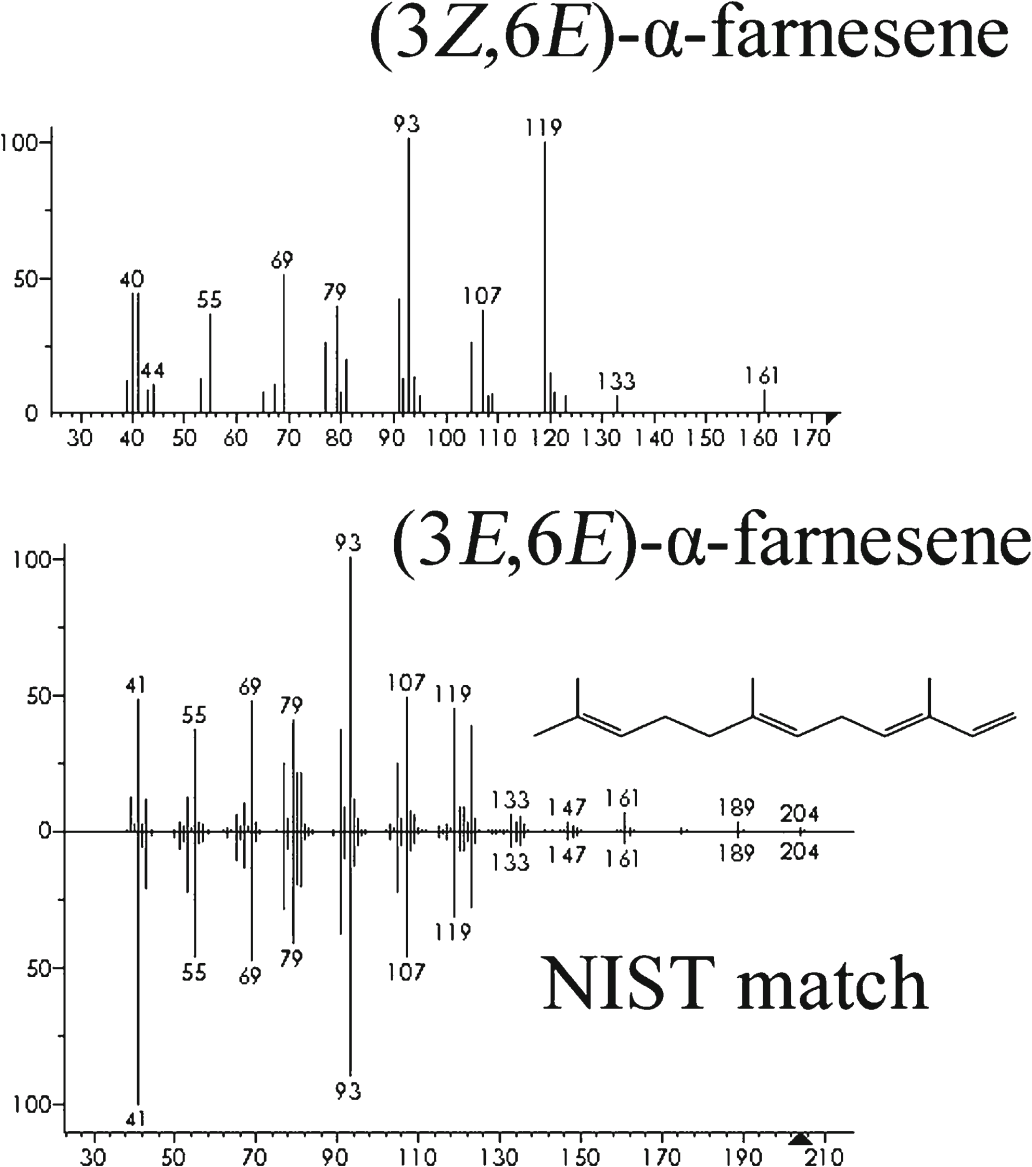
EI mass spectra of a synthetic standard of (3*Z*,6*E*)-α-farnesene (*upper*), synthetic standard of (3*E*,6*E*)- α-farnesene (*middle*), and NIST library match for (3*E*,6*E*)-α-farnesene (*lower*)

In 2013, using the Solarmax T5 lighting system, we observed an increase in the abundance of the unidentified compound in several samples of headspace volatiles collected from male beetles (Fig. 3). The EI-mass spectrum (Fig. 4) of the unknown compound closely matched that of an isomer of α-farnesene (NIST version 2.0, 2002), in particular, the (3*E*,6*E*)-α-farnesene

To identify the antennally active isomer of α-farnesene, we compared synthetic (3*Z*,6*E*)-α-farnesene and (3*E*,6*E*)-α-farnesene *via* GC/MS (Figs. 3 and 4). The (3*E*,6*E*)-α-farnesene had an identical retention time (15.13) to that of the unknown compound in the male aeration (Fig. 3). Both the synthetic (3*E*,6*E*)-α-farnesene and the unknown compound in the male aeration had identical Kovats indices of 1511 on a DB-5 column. Adams (1995) reported a Kovats index of 1508 for this compound on a DB-5 column.

**Fig. 5:**
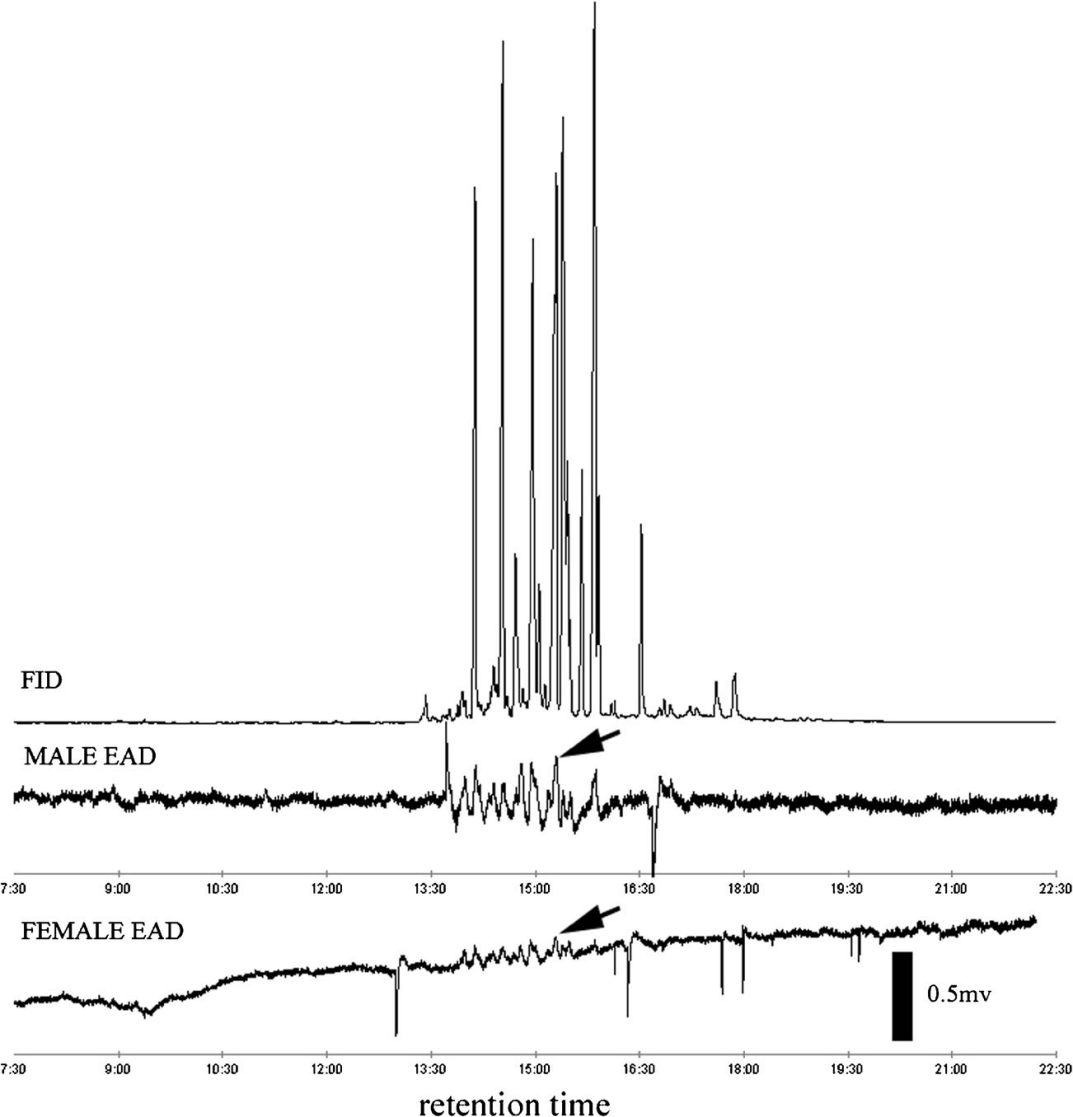
Typical GC/EAD responses of adult male and female *Anoplophora glabripennis* to a commercially-available mixture injection that consisted of 2000 ng **Sigma Aldrich farnesene isomers** in 1 ul hexane

Multiple GC/EAD responses were consistently observed to components of the commercial mixture of farnesene isomers (RT 13–18 min) from both female and male beetles (Fig. 5).

**Fig. 6:**
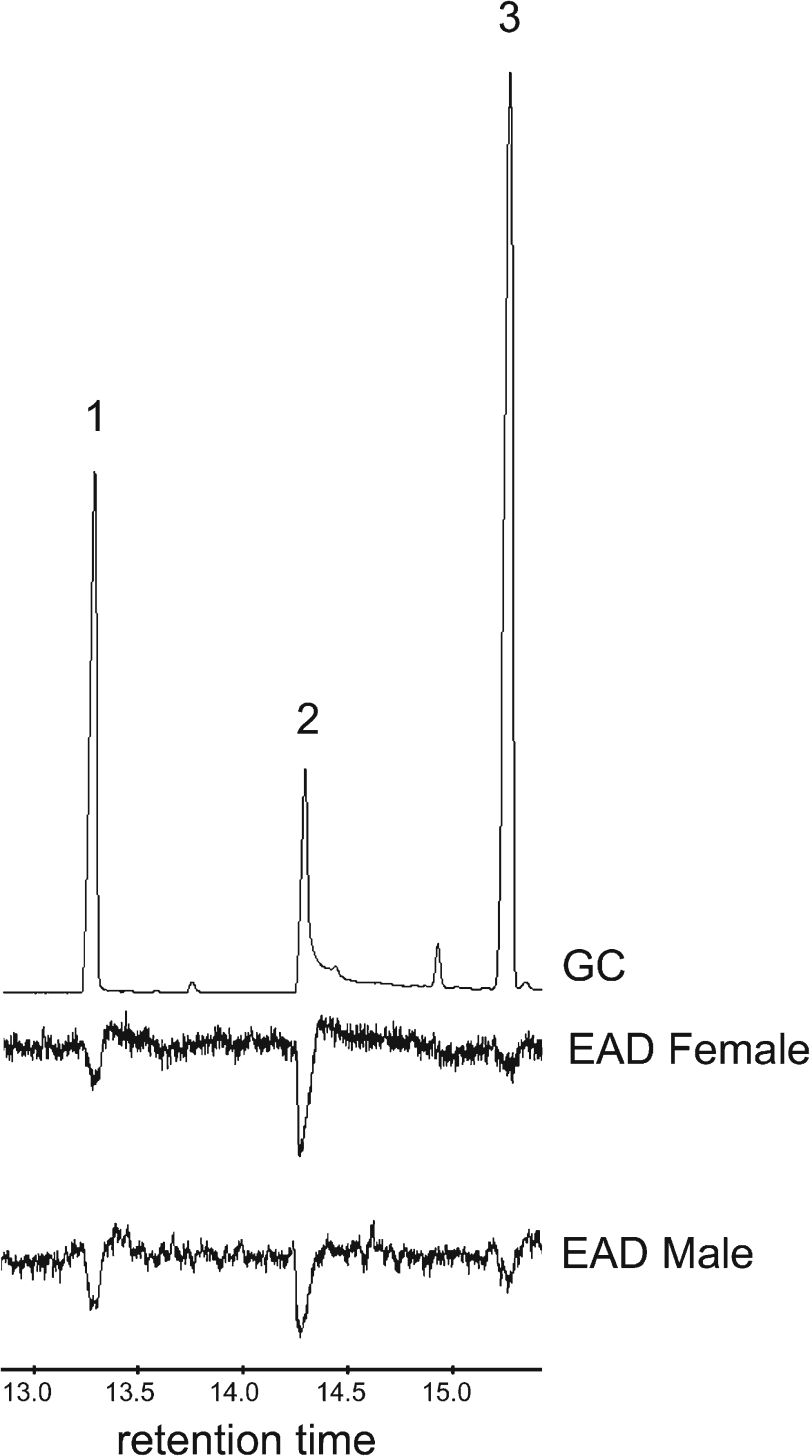
GC/EAD responses of adult female (18-day-old) and male (21-day-old) *Anoplophora glabripennis* to a synthetic mix of 200 ng each of (*1*) 4-(*n*-heptyloxy)butanal, (*2*) 4-(*n*-heptyloxy)butan-1-ol./ and (*3*) (3*E*,6*E*)- α-farnesene

Both male and female *A. glabripennis* antennae gave consistent GC/EAD responses to a synthetic mix of 4-(*n*-heptyloxy)butanal, 4-(*n*-heptyloxy)butan-1-ol, and (3*E*,6*E*)-α-farnesene (Fig. 6).

### Olfactometer Assays

Both male and female *A. glabripennis* exhibited significant positive taxis toward (3*E*,6*E*)-α-farnesene when it was tested alone against a hexane control (*N* = 25) (Table 1). Females showed non-significant 56 and 52 % positive responses to 1 and 10 μg doses of (3*E*,6*E*)-α-farnesene, but a significant 76 % positive response when a 20 μg dose was tested. Males exhibited significant positive taxis at 1 and 10 μg doses of (3*E*,6*E*)-α-farnesene (76 and 72 %, respectively) but a non-significant 32 % response to 20 μg. Males did not show a significant attraction (48 %) to a 10 μg dose of mixed farnesene isomers.

**Table 1:** Two-choice olfactometer behavioral assays comparing responses of male and female *Anoplophora glabripennis* (*N* = 25) to different doses of (3*E*,6*E*)-α-farnesene *vs.* a hexane control (*X* ^2^ analysis)

Sex of beetle tested	Odor source 1	Odor source 2	Choice 1	Choice 2	% response to choice 1	X^2^	*P*
	((3*E*, 6*E*)-α-farnesene)	(hexane)					
Female	1 μg	1 μl	14	11	56 %	0.16	0.921
Female	10 μg	10 μl	13	12	52 %	0.00	NS
Female	20 μg	20 μl	19	6	76 %	5.76	0.0093
Male	1 μg	1 μl	19	6	76 %	5.76	0.0093
Male	10 μg	10 μl	18	7	72 %	4.00	0.0269
Male	20 μg	20 μl	8	17	32 %	2.56	0.0693
Male	10 μg mixture of isomers	10 μl	12	13	48 %	0.00	NS

Female responses to the two known male-produced pheromone components were increased by addition of (3*E*,6*E*)-α-farnesene (Table 2). When a 1:1 blend of 4-(*n*-heptyloxy)butan-1-ol and 4-(*n*-heptyloxy)butanal (1 ug each) was presented to females against a hexane control, a 44 % non-significant response to the stimulus was observed. When 1 μg of (3*E*,6*E*)-α-farnesene was added to the 1:1 pheromone blend, female response increased to a significant 72 % positive response over the hexane control. When the dose of 4-(*n*-heptyloxy)butan-1-ol and 4-(*n*-heptyloxy)butanal (1:1 mix) was increased to 10 μg and presented to females (against a hexane control), a 48 % response to the stimuli was observed. The response of female beetles to the two-component pheromone blend was seemingly improved upon the addition of 10 μg of (3*E*,6*E*)-α-farnesene (64 %) although the response was not significant.

**Table 2:** Two-choice olfactometer behavioral assays comparing responses of female *Anoplophora glabripennis* (*N* = 25) to blends of 4-(*n*-heptyloxy)butan-1-ol and 4-(*n*-heptyloxy)butanal with and without (3*E*,6*E*)-α-farnesene (*χ* ^2^ analysis)

Sex of beetle tested	Odor source 1: 4-(*n*-heptyloxy)butan-1-ol + 4-(*n*-heptyloxy)butanal + (3*E*,6*E*)-α-farnesene (μg)	Odor source 2: hexane (μl)	Choice 1	Choice 2	% response to choice 1	*χ* ^2^	*P*
Female	1 μg + 1 μg + 0 μg	2 μl	11	14	44 %	0.16	0.921
Female	1 μg + 1 μg + 1 μg	3 μl	18	7	72 %	4	0.027
Female	10 μg + 10 μg + 0 μg	20 μl	12	13	48 %	0	NS
Female	10 μg + 10 μg + 10 μg	30 μl	16	9	64 %	1.44	0.162

Male responses to the two known male-produced pheromone components improved upon the addition of (3*E*,6*E*)-α-farnesene (Table 3). When a 1:1 blend of 4-(*n*-heptyloxy)butan-1-ol and 4-(*n*-heptyloxy)butanal (1ug each) was presented to males against a hexane control a 52 % non-significant response to the stimulus was observed. When a 1 μg amount of (3*E*, 6*E*)-α-farnesene was added to the 1:1 pheromone blend, male response increased to a significant 76 % positive response over the hexane control. When presented with a higher dosage of 10 μg per component, males did not show a significant response to either the 1:1 blend of pheromone components or the 1:1:1 mixture with (3*E*,6*E*)-α-farnesene (Table 3).

**Table 3:** Two-choice olfactometer behavioral assays comparing responses of male *Anoplophora glabripennis* (*N* = 25) to blends of 4-(*n*-heptyloxy)butan-1-ol and 4-(*n*-heptyloxy)butanal with and without (3*E*,6*E*)-α-farnesene (*χ* ^2^ analysis)

Sex of beetle tested	Odor source 1: 4-(*n*-heptyloxy)butan-1-ol +4-(*n*-heptyloxy)butanal + (3*E*,6*E*)-α-farnesene (μg)	Odor source 2: hexane (μl)	Choice 1	Choice 2	% response to choice 1	*χ* ^2^	*P*
Male	1 μg + 1 μg + 0 μg	2 μl	13	12	52 %	0	NS
Male	1 μg + 1 μg + 1 μg	3 μl	19	6	76 %	5.76	0.0093
Male	1 μg + 1 μg + 0 μg	20 μl	12	13	48 %	0	NS
Male	1 μg + 1 μg + 1 μg	30 μl	8	17	32 %	2.56	0.0693

## Discussion

The results provide evidence that (3*E*,6*E*)-α-farnesene is a potential third component of the male-produced aggregation pheromone that may play a role in the mating behavior and complex chemical ecology of *A. glabripennis*. We have never observed (3*E*,6*E*)-α-farnesene in aerations from either fed or unfed female *A. glabripennis* or from twig cuttings of striped maple (data not shown), and the compound is thus assumed to be male-specific. The compound was attractive to both male and female beetles in an olfactometer bioassay and could enhance the attractiveness of the two pheromone components identified previously, 4-(*n*-heptyloxy)butan-1-ol and 4-(*n*-heptyloxy)butanal. More testing is planned to confirm field activity, and to determine optimum release rates of these three male-produced compounds.

Within the Cerambycidae, the majority of pheromones identified to date are produced by males and attract both sexes (Hanks and Millar 2013). This is particularly true in the subfamily Lamiinae, of which *A. glabripennis* is a member (Allison et al. 2012; Fierke et al. 2012; Fonseca et al. 2010; Mitchell et al. 2011; Pajares et al. 2010; Teale et al. 2011). In the last decade, research has revealed that there is substantial pheromonal parsimony within the Cerambycidae (Hanks and Millar 2013). In the Lamiinae, male *Monochamus galloprovincialis* (Olivier) (Pajares et al. 2010), *M. alternatus* (Hope) (Teale et al. 2011) and *M. sutor* L. (Pajares et al. 2013) all produce 2-undecyloxy-1-ethanol as an aggregation pheromone. The structure of this is similar to the dialkyl ethers 4-(*n*-heptyloxy)butan-1-ol and 4-(*n*-heptyloxy)butanal produced by *A. glabripennis* (Zhang et al. 2002) and to 2-(4-heptyloxy-1-butyloxy)-1-ethanol, produced by male *M. leuconotus* (Pascoe) (Hall et al. 2006).

The use of terpenoids as a male produced aggregation pheromone has been reported by Lacey et al. (2008) for the cerambycine species *Megacyllene caryae* (Gahan). A blend of alkanoids, terpenoids, and aromatic alcohols was found to be a general aggregation pheromone for this species in olfactometer studies. Mitchell (2012) reported that male *Megacyllene robiniae* (Forster) produced six terpenoids (not produced by females), which may be minor pheromone components. One of the compounds identified by Mitchell (2012) was *β*-farnesene.

α-Farnesene was first reported as a bioactive volatile released from the wax of apple skin (Huelin and Murray 1966: Murray and Huelin 1964). Both the (3*E*,6*E*) and (3*Z*,6*E*) isomers of α-farnesene have been found to increase calling and oviposition rates in codling moth, *Cydia pomonella* (L.) females (Yan et al. 2003). Electrophysiological responses of antennae to (3*E*,6*E*)-α-farnesene and (3*Z*,6*E*)-α-farnesene have been reported for a number of insect species (Cha et al. 2008; Rodriguez-Saona et al. 2006; Tasin et al. 2005; Wie and Kang 2006; Yarden et al. 1996). Isomers of α-farnesene have been linked to behavioral responses such as taxis (Cha et al. 2008; Landolt et al. 2000; Silk et al. 2010; Wie and Kang 2006), oviposition (Sutherland et al. 1977), and alarm calling (Šobotnik et al. 2008).

Silk et al. (2010) found that farnesene isomers may play a role in the chemical ecology of the cerambycid, *Tetropium fuscum*. There is strong evidence to show that stress-induced sesquiterpene components of conifers, including farnesene, may play a role in enabling *Tetropium fuscum* to locate a weakened host (Silk et al. 2010). Silk et al. (2010) found that *T. fuscum* gave a consistent GC/EAD response to synthetic (3*Z*,6*E*)-α-farnesene, which matched the GC retention time of the antennally active unknown sesquiterpene in spruce oil. An antennal response to (3*E*,6*E*)-α-farnesene also was observed in a small number of replicates but was lower in amplitude (Silk et al. 2010). Our electrophysiological recordings for male and female *A. glabripennis* showed that they also gave multiple antennal responses to isomers of farnesene in a mixture of isomers, although they were not observed in male aerations. These other antennally-active farnesene isomers could be repellant to adult beetles, and may prevent this readily-available mixture of isomers being a viable lure option. In our preliminary olfactometer test, 10 μg of the mixture of farnesene isomers were not attractive to males. The basic ‘structural skeleton’ of farnesene may elicit an antennal response in *A. glabripennis,* but the specific (3*E*,6*E*)-α-farnesene appears to be necessary for a behavioral response. The (3*E*,6*E*)-α-farnesene isomer appears to be a pheromone component for the Mediterranean fruit fly *Ceratitis capitata* (Wied.) (Heath et al. 1991), and recently has been identified as being a male-produced pheromone component of the banana-spotting bug, *Amblypelta lutescens lutescens* Distant (Heteroptera: Coreidae) by Khrimian et al. (2012).

Our identification of an α-farnesene isomer as a potential attractant for *A. glabripennis* supports findings of previous studies done on another *Anoplophora* species. Volatiles that have been implicated in short- and long-distance mate location in the citrus longhorned beetle, *Anoplophora malasiaca* (referred to subsequently as *A. chinensis*; see Lingafelter and Hoebeke 2002) include the sesquiterpenes *β*-elemene, *β*-caryophyllene, α-humulene, (3*E*,6*E*)-α-farnesene, and several unidentified compounds (Yasui et al. 2007, 2008). The laboratory and field assays done by Yasui et al. (2007) show that both male and female *A. chinensis* use sesquiterpenes such as (3*E*,6*E*)-α-farnesene for mate location over both short and long distances. Adachi (1990) differentiated small- and large-scale movements for *A. chinensis*. The former consisted of walking bouts interspersed with brief flights between trees. The latter comprised walking on the ground and flying longer distances. After using sesquiterpenes in field tests, Yasui et al. (2007) concluded that sesquiterpenes were more important for attraction over a longer distance. After landing in the vicinity of the odor source, adults located mates using shorter range olfactory cues. The final approach then was made by visual and olfactory cues (Fukaya et al. 2005a, b) before direct antennal contact/reception of the female-produced contact pheromone (Fukaya et al. 1999).

A similar scenario for mate location in *A. glabripennis* also has been suggested in which *A. glabripennis* utilizes male- and female-produced long range pheromones to find a mate among a forest of host odors (Hoover et al. 2014; Nehme et al. 2010; Wickham et al. 2012). Li et al. (1999) suggested that a female-produced contact pheromone played a role in the final stages of mate recognition. Zhang et al. (2003) compared male and female body washes of adults and identified five monounsaturated hydrocarbons that elicited copulatory behavior immediately after antennal contact. Female *A. glabripennis* also have been found to utilize a four component, sex specific trail pheromone (Hoover et al. 2014). These components were identified as 2-methyldocosane and (*Z*)-9-tricosane (major components) as well as (*Z*)-9-pentacosene and (*Z*)-7-pentacosene (minor components).

Identification of ‘long range’ attractants is essential for an effective field lure, and both female- and male-produced compounds have been examined as candidate long-range attractants for *A. glabripennis*. Wickham et al. (2012) tested the hypothesis that one or more female-produced contact sex pheromones were precursors that underwent abiotic oxidation to yield more volatile, longer-range pheromone components. Males were found to be preferentially attracted to ozonized female body washes over crude body washes. In GC/EAD analyses, three antennally-active aldehydes, heptanal, nonanal, and hexadecanal were detected. In field tests on traps in China, combinations of these aldehydes with linalool oxide and other host kairomones captured more beetles than controls, and captured significantly more males. Wickham et al. (2012) proposed that females select host trees and after a period of feeding, release volatiles that attract males. They hypothesized that the two known male-produced pheromones acted over a shorter range once males had been attracted to the host tree. If this behavioral scenario is correct, then our newly identified component potentially could increase this short range attraction of females.

Our results show that by itself, (3*E*,6*E*)-α-farnesene is attractive to both male and female *A. glabripennis* at varying doses. Males appear to show significant responses in assays to lower doses, which may indicate that (3*E*, 6*E*)-α-farnesene is being used as a general aggregation pheromone but is repellent and dispersive to males at higher concentrations. Females appeared to be more responsive to (3*E*,6*E*)-α-farnesene at higher doses. The other two previously identified male pheromones, 4-(n-heptyloxy)butan-1-ol and 4-(n-heptyloxy)butanal were not attractive individually at 1 μg doses to male or female *A. glabripennis* in olfactometer tests (Nehme et al. 2009). Virgin females, but not virgin males, showed a significant olfactometer response to a 1 μg : 1 μg combination of the two components (Nehme et al. 2009). Female responses to these two components appeared to be enhanced by host plant kairomones, and field tests have shown statistically significant but limited attraction (Meng et al. 2014; Nehme et al. 2010). This suggested that other chemical cues or signals may be involved in improving the likelihood of finding a mate (Hoover et al. 2014). We did not see significant attraction of male or female *A. glabripennis* to a 1:1 mix of 4-(*n*-heptyloxy) butan-1-ol and 4-(*n*-heptyloxy) butanal at either the 1 μg : 1 μg or 10 μg :10 μg dosages in any of our olfactometer tests. Significant attraction was observed only upon the addition of the (3*E*,6*E*)-α-farnesene (1:1:1) for females at the 10 μg dose. Further research is needed to determine if (3*E*, 6*E*)-α-farnesene is a third component of a male produced pheromone blend, or if it is a general aggregation pheromone that is effective by itself.
